# Video studies of passage by *Anopheles gambiae* mosquitoes through holes in a simulated bed net: effects of hole size, hole orientation and net environment

**DOI:** 10.1186/s12936-015-0713-4

**Published:** 2015-05-13

**Authors:** James Sutcliffe, Kathryn L. Colborn

**Affiliations:** Department of Biology, Trent University, Peterborough, ON K9J 7B2 Canada; Entomology Branch, US Centers for Disease Control and Prevention, Atlanta, GA 30341 USA; Graduate Group in Biostatistics, University of California, Berkeley, CA USA

**Keywords:** *Anopheles*, Mosquito behaviour, Host-seeking, Hole passage, Bed nets, ITN, LLIN

## Abstract

**Background:**

Holes in netting provide potential routes for mosquitoes to enter ITNs. Despite this, there is little information on how mosquitoes respond to holes in bed nets and how their responses are affected by hole size, shape and orientation or by ambient conditions around the net.

**Methods:**

Female *Anopheles gambiae* (G3) were recorded in a simulated bed net consisting of two sizes of untreated netting-covered behavioural arenas placed above and beside (to simulate the bed net roof and sides respectively) the experimenter who was a source of host cues from ‘inside’ the net. A round hole of 9 mm or 13 mm diameter was cut into the centre of the netting of each arena. Videos of unfed female mosquitoes in arenas were analysed for time spent flying, walking and standing still and for exit through the hole. The effects of the experimenter on temperature and relative humidity around the simulated net were also measured.

**Results:**

Mosquitoes were significantly more active in overhead arenas than in arenas to the side. Hole passage was significantly more likely in smaller arenas than larger ones and for larger holes than smaller ones. In arenas to the side, hole passage rate through small holes was about 50 % less likely than what could be explained by area alone. Passage rate through holes in overhead arenas was consistent with hole area. Temperature in arenas did not strongly reflect the experimenter’s presence in the simulated net. Relative humidity and absolute humidity in overhead arenas, but not in arenas to the side, were immediately affected by experimenter presence.

**Conclusions:**

Higher levels of activity in overhead arenas than in arenas to the side were likely due to the rising heat and humidity plume from the experimenter. Lower than expected passage rates through smaller vertically oriented holes may have been be due to an edge effect that does not apply to horizontally oriented holes. Results suggest that current methods of assessing the importance of physical damage to ITNs may not accurately reflect mosquito entry risk in all cases.

## Background

Insecticide-treated bed nets (ITNs) have been credited with reducing the global burden of malaria since mass distribution programmes began in the early 2000s [1]. ITNs are effective because they are both a physical and chemical barrier to host-seeking anopheline mosquitoes which otherwise might have free access to human hosts at night. Holes in the netting of bed nets provide a potential route for malaria-infected mosquitoes to enter the net and bite. This could result in malaria infection for the net occupants or, if the occupants are already infected, it could result in infection of mosquitoes and the possibility of subsequent transmission to other human victims. ITNs can sustain significant levels of physical damage and still provide the equivalent protection of an undamaged untreated net because of the insecticide ‘back up’ [2, 3]. Indeed, this is an essential element of the ITN strategy. In areas of insecticide resistance [4], however, or where insecticide levels in the net material have been sufficiently depleted, or where damage to the net is extensive [5], this back up protection may not be effective, thus raising the risk of mosquito entry into damaged nets. The potential significance of physical damage on ITN effectiveness is further elevated by the fact that several studies have found that ITNs are deteriorating faster than the 3–5 year lifespan originally anticipated [6–8]. Given this, several recent studies have focussed on the causes and characteristics of bed net damage. Holes in ITNs come about in a number of ways including by chewing animals, burns from open flames and by snagging on sharp objects [9, 10]. Degrees of damage vary widely in ITNs in the field. Smith *et al*. [10] showed that over 50 % of 2023 holes (not including holes 0.5 cm across or less) in 50 nets taken from the field after 38 months of use had long dimensions of 3 cm or less while 31 holes greater than 10 cm were found.

While it is obvious that holes in bed nets provide a potential way in for mosquitoes, there is very little understanding of how mosquitoes interact with holes in bed nets when they come across them or how they pass through them and into the net. There are several dimensions to this question. For instance, how is the probability of hole passage affected by bed net holes of different sizes (since holes may range from a few millimetres to tens of centimetres in diameter) and shapes? Additionally, holes may occur anywhere on the net. Is a hole of a given shape and size on the roof of a bed net as likely to admit mosquitoes encountering it as the same hole on the sides or ends of the net? Finally, how does the environment around the net, which is a product of ambient environmental conditions and the odour, moisture and heat produced by the bed net occupants affect mosquito hole passage behaviour and are these effects the same on all parts of the net or do they vary with location on the net?

To investigate these questions, this study used video cameras to record individual mosquito interactions with holes of different sizes in a simulated bed net. For practical purposes and because a large proportion of bed net holes are small [10], the recordings were done with two sizes of small hole. Specifically, mosquito behaviour and hole passage rates were determined for a 9 mm diameter or 13 mm diameter round hole in both small (85 mm dia.) and large (170 mm dia.) behavioural arenas placed above the experimenter (to simulate the bed net roof) or beside the experimenter (to simulate the bed net side or end). Measurements were also made around the simulated net of how heat and humidity from a simulated bed net occupant affects conditions on different locations of the simulated bed net.

## Methods

### Source colonies

Mosquitoes used for the videos were drawn from stock colonies of *Anopheles gambiae s.s.* (G3 strain) maintained by the Malaria Branch at the Centers for Disease Control and Prevention (CDC) in Atlanta, Georgia. Colonized larvae, pupae and adults were maintained at 28 °C on a 12 h:12 h light:dark cycle with an 30 min artificial sunrise and sunset. Adults emerged directly into 4 L cylindrical cardboard containers and were provided with carbohydrates *ad libitum* in the form of 10 % corn syrup in water.

Video recordings were made under subdued room lighting conditions and used eight day-old nulliparous, not previously blood-fed females. Two hours before experiments began, an appropriately aged cohort of approximately 50 female mosquitoes was placed in a half litre mesh-covered cardboard holding cage. This cage was kept outside the environmental chamber where recordings were done and away from contact with potential host stimuli.

### Experimental set-up

Experiments were performed in a simulated untreated bed net set up in a 5.5 m × 2.8 m × 2.5 m high environmental chamber in the insectary facilities. General conditions in the chamber were maintained at approximately 26 °C and 60 % RH. Room fans were shut off to minimize air turbulence.

**Fig. 1:**
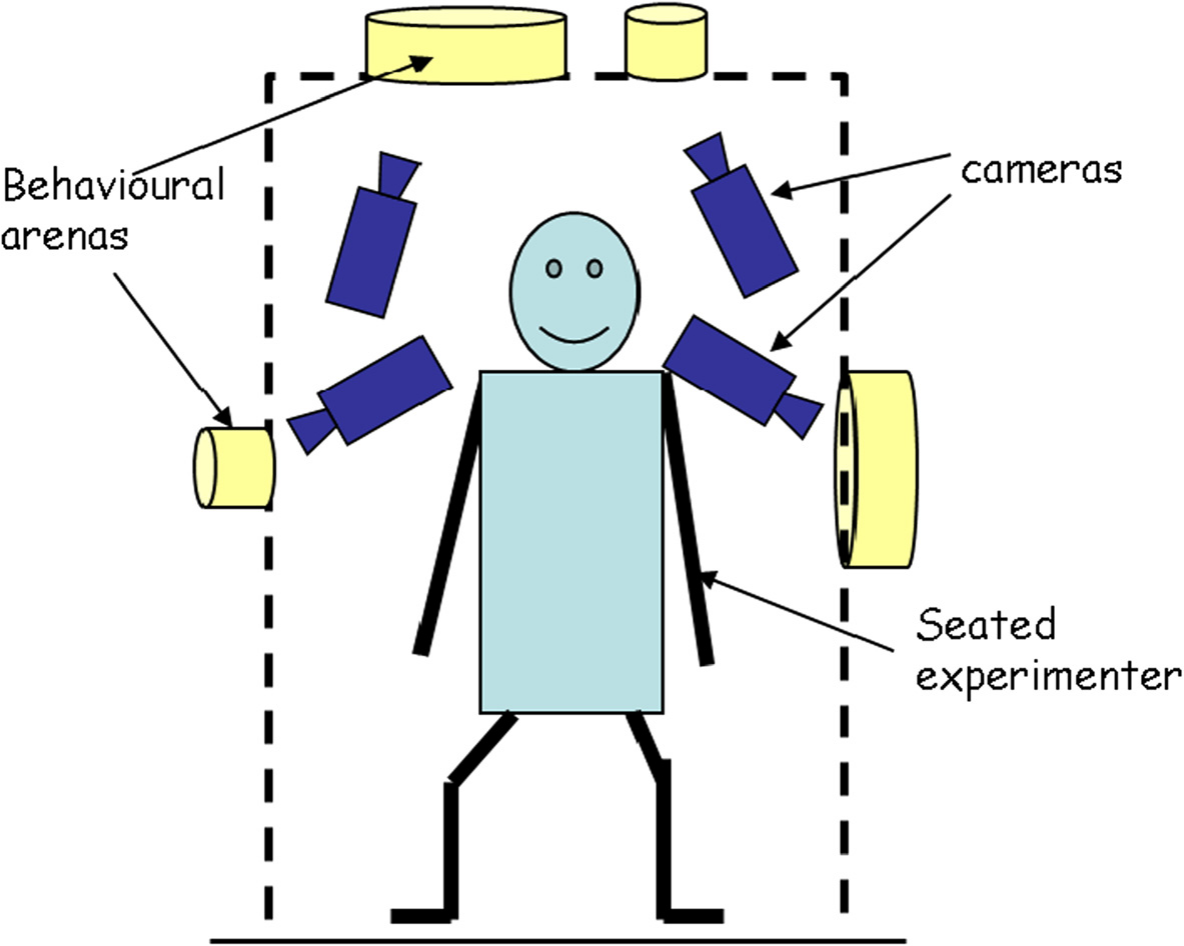
Diagram of positions of behavioural arenas and cameras on virtual bed net (dashed line)

**Fig. 2:**
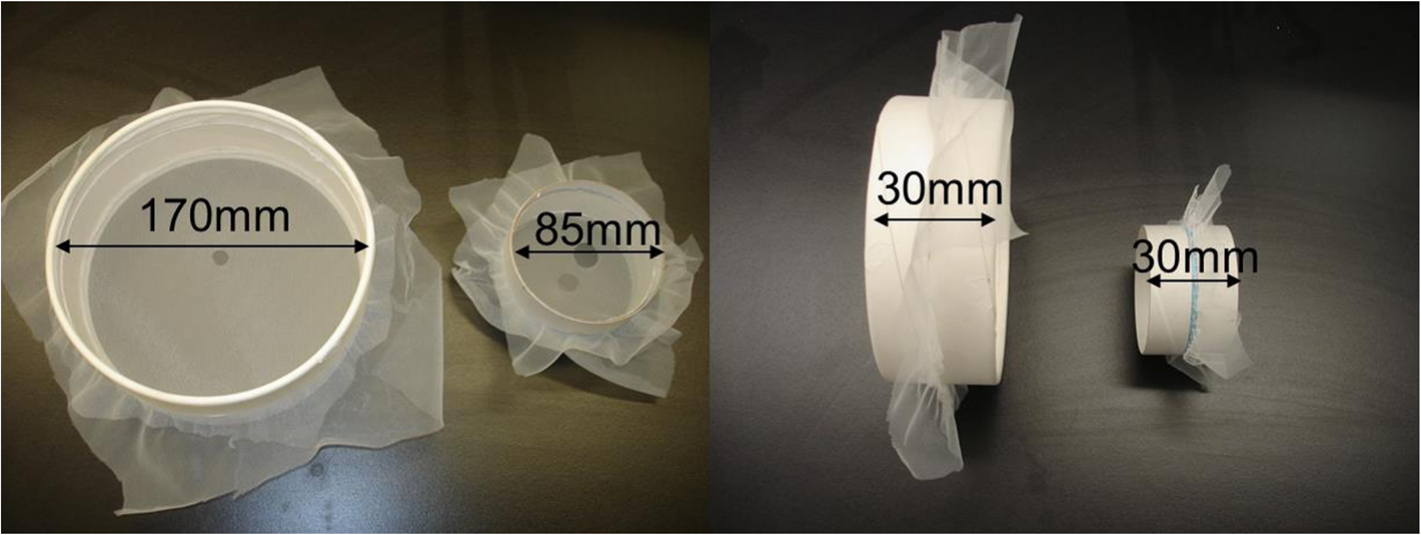
Front (left panel) and side (right panel) views of behavioural arenas of two sizes. Larger arena (left in left panel) with a 9 mm diameter hole. Smaller arena (right in left panel) with a 13 mm hole

**Fig. 3:**
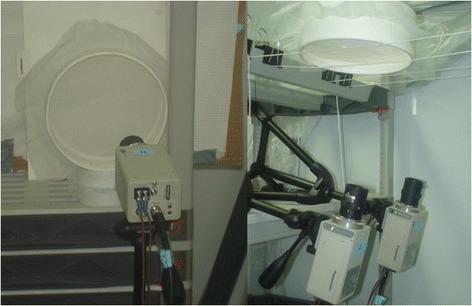
Video cameras positioned in front of vertically oriented arena (left panel) and below horizontally oriented arenas (right panel)

The simulated bed net consisted of netting-covered behavioural arenas placed on a shelf approximately 35 cm to the side of the seated experimenter (who filled the role of the bed net occupant) or supported approximately 35 cm above the experimenter’s head (Fig. 1). Arenas (Fig. 2) were made from cut down cylindrical cardboard containers. Two sets of four arenas (8 arenas in total) were made. Arenas from one set were placed above the experimenter with the netting oriented horizontally to simulate the bed net roof. Arenas from the other set were placed about 35 cm away and to the side of and facing the experimenter with the netting oriented vertically to simulate the side of the bed net. Each set consisted of two arenas with a diameter of 170 mm and two with a diameter of 85 mm. All arenas were 30 mm deep. The side-simulating (vertically oriented) arenas were covered with untreated polyester tulle (approx. 1.5 mm mesh size) netting on both ends. The top-simulating horizontally oriented arenas were covered with the same type of netting on the bottom side nearer to the experimenter. To reduce resistance to rising heat and humidity from the experimenter, the top side was covered with a more open (approx. 4 mm mesh size) netting. One 170 mm diameter arena and one 85 mm arena in each set had a 13 mm diameter round hole cut in the approximate center of the netting facing the experimenter. The other arenas in each set had a 9 mm diameter round hole cut in the corresponding location. To ensure holes were as regular and circular as possible, they were cut with Irex® scissors using a paperboard template attached to the underside of the netting as a guide. The holes were examined with the aid of a dissecting microscope to ensure their edges were clean and free of potentially encumbering fibers. The set-up was completed by up to four video cameras (Panasonic model WV-BP334) (Figs. 1, 3), one for each arena. The cameras were connected to a portable cart-mounted computer capable of recording up to four video streams simultaneously using Noldus Recorder® software (Noldus Information Technology, Wageningen, The Netherlands).

### Experimental procedure

Experiments were done in three sessions. Each session consisted of several recording runs involving two arenas at a time set up beside (session 1) or above (session 2) the experimenter, or four arenas at a time (session 3), two above and two beside the experimenter (Table 1). To start a recording run, a single female mosquito was placed in each arena according to a pre-determined rotation designed to test each arena size and hole size combination equally in any given session. The arenas were then placed in front of or above their respective cameras. The experimenter took up position seated in the simulated bed net in front of the monitoring computer (Fig. 1). Mosquitoes were given 30 s to acclimate to the arena after which the recording was started. In the few cases where mosquitoes exited from their arenas during the acclimation period, the run was completed with the remaining mosquitoes. During the recording run the experimenter was careful not to breathe directly into the arenas or to make sudden movements that might be seen or otherwise detected by the mosquitoes. Each arena was recorded for 10 min. Mosquitoes were not re-used. In the combined three recording sessions the target was 10 or more passages (i.e. entries into the simulated net) for each arena size, hole size and orientation combination. This was achieved for all combinations except the 170 mm arena/9 mm hole/vertical orientation combination for which there were nine passages.

**Table 1:** Summary of outcomes of recording sessions 1-3

a) Vertical orientation (beside experimenter)		Small arena (85 mm dia.)		Large arena (170 mm dia.)		
		Hole diameter		Hole diameter		
Recording session	# mosquitoes	9 mm	13 mm	9 mm	13 mm	Total
1	Tested	42	29	40	31	142
	Showing no activity	5	1	4	3	13
	Passing through hole	9	16	5	9	39
2	Tested	-	-	-	-	-
	Showing no activity	-	-	-	-	-
	Passing through hole	-	-	-	-	-
3	Tested	25	26	30	24	105
	Showing no activity	1	5	4	5	15
	Passing through hole	10	15	4	11	40
Total	Tested	67	55	70	55	247
	Showing no activity	6	6	8	8	28
	Passing through hole	19	31	9	20	79
b) Horizontal orientation (above experimenter)		Small arena (85 mm dia.)		Large arena (170 mm dia.)		
		Hole diameter		Hole diameter		
Recording session	# mosquitoes	9 mm	13 mm	9 mm	13 mm	Total
1	Tested	-	-	-	-	-
	Showing no activity	-	-	-	-	-
	Passing through hole	-	-	-	-	-
2	Tested	11	9	17	12	49
	Showing no activity	0	0	0	0	0
	Passing through hole	7	7	8	11	33
3	Tested	21	28	28	26	103
	Showing no activity	0	0	0	1	1
	Passing through hole	13	23	12	8	56
Total	Tested	33	37	45	38	153
	Showing no activity	0	0	0	1	1
	Passing through hole	20	30	20	19	89

See text for a description of each session. a) Vertically oriented arenas (arenas located beside the experimenter) and b) horizontally oriented arenas (arenas located above the experimenter). Dashes in a cell indicate no recordings were made for that combination in that session

### Measurements of ambient conditions in arenas

Temperature and relative humidity conditions were measured in the arenas around the simulated bed net in a separate session with no mosquitoes present using Hobo® data loggers (Onset Technologies, model U12). One logger was placed with its sensor window down on the netting inside a 170 mm diameter arena placed in the overhead position (bed net roof simulating position) and one was placed with its sensor window facing into the simulated bed net in the side simulating position. A third (reference) logger was placed approximately 3.5 m away from the simulated net on a shelf 1.2 m above the floor. Loggers were synchronized to record once per second throughout the test which consisted of three cycles. In each cycle, the simulated net was occupied by the experimenter for 10 min and then left unoccupied for three minutes during which time the experimenter stood off to the side but did not leave the room. A five-minute period during which the simulated bed net was unoccupied preceded the first of the three cycles.

Recordings were downloaded from the recording units using HOBOware Pro® software and data were processed using Microsoft® Excel. Hoboware conversion software was used to calculate second-by-second absolute humidity values from temperature and relative humidity data for each recorder location.

### Behavioural and statistical analysis of videos

All videos were analysed using Noldus Observer™ (version XT 8.0) software (Noldus Information Technology, Wageningen, The Netherlands). Behaviours of mosquitoes in each recording were classified as one of three mutually exclusive state events: ‘flying’, ‘walking’ and ‘still’. ‘Flying’ was scored any time the mosquito was beating its wings and moving in the arena. This included airborne flight and skimming over the mesh surface during which the mosquito often made repeated contact with the net. ‘Walking’ was scored as any displacing activity in the arena that could not be scored as ‘flying’. Because walking could be very slow, it was sometimes difficult to draw the line between it and the ‘still’ state. ‘Walking’ could also be more vigorous and include momentary bouts of wing beating (but no lift off from the net) and probing with the mouthparts through the net mesh. The ‘still’ state was scored any time the mosquito was standing in one place in the arena. Often mosquitoes were completely still in this state though sometimes they would clean. Hole passage was scored as a point event defined by the mosquito passing through the hole in the netting. If hole passage did not occur within 10 min, the analysis was terminated.

Results of the Observer video analyses were exported to an Excel spreadsheet and analysed in R [11]. Kaplan-Meier survival functions were fit to the observed flying times in the arenas for each mosquito using the survival package [12]. Mosquitoes that were not active (had zero flight times, n = 29, 7 %) were excluded from the survival analyses. To test the ratios of the rates of passage for the various arena configurations conditional on orientation, Cox Proportional Hazards regression models [13] were fitted to the data. Fixed factors for arena size and hole size were included in separate models for vertically oriented arenas and horizontally oriented arenas, and tied flight times were handled by Efron’s method [14].

## Results

### Effects of hole diameter and orientation on hole passage

Three hundred and ninety nine video recordings were made in the course of the three sessions; 247 (61.9 %) in the vertical orientation and 152 (38.1 %) in the horizontal orientation (Table 1).

There were clear differences between activity levels of mosquitoes in differently oriented arenas (Table 2). In vertically oriented arenas (beside the experimenter), 28 (11.3 %) mosquitoes were completely inactive for the entire 10 min while in horizontally oriented arenas (above the experimenter) only one (0.7 %) mosquito showed no activity for the entire 10 min. Total flight durations were positively skewed for all combinations of arena and hole size in both orientations (Table 2). Mosquitoes in horizontally oriented arenas of both sizes exhibited more overall flight activity (Table 2, median flight duration = 93.0 s [40, 198]) than mosquitoes in vertical arenas (Table 2, median flight duration = 48.5 s [12, 117.5). This difference was significant according to a Mann–Whitney-Wilcoxon test (*p* < 0.001). Median total flight times prior to hole passage were significantly shorter for mosquitoes in vertically oriented arenas compared to mosquitoes in horizontally oriented arenas (Table 2, *p* = 0.01). Mosquitoes that did not pass through the hole in the netting spent significantly more time flying in the horizontally oriented arenas compared to the vertically oriented arenas (Table 2, *p* < 0.001). In both arena orientations, mosquitoes tended to fly closer to the netting on the experimenter’s side of the arenas and tended to fly across the netting while facing it and bumping or touching it repeatedly. Compared to flights in the vertically oriented arenas, flights in the horizontally oriented arenas appeared to be closer to the netting, to be more energetic, to involve more contact with the netting and to be punctuated by more short periods of walking. Flight paths in the horizontally oriented arenas were also characterized by frequent tight turns which, compared to flight paths in vertically oriented arenas, resulted in less translational movement across the netting per unit of flight time. When walking on the netting of the horizontally oriented arenas mosquitoes would often attempt to probe pressing their heads and mouthparts through the mesh.

**Table 2:** Flight activity (including 25th and 75th percentiles) of mosquitoes that passed and that did not pass through holes by arena orientation irrespective of hole size and arena size (including mosquitoes that were inactive)

Arena orientation	Passed through hole		Did not pass through hole		Total
	Median flight time (25th,75th)	N (row %)	Median flight time (25th,75th)	N (row %)	Median flight time (25th,75th)
Horizontal	75 (31, 131)	89 (59)	167 (79, 371)	63 (41)	93 (40, 198)
Vertical	40 (20, 103)	79 (32)	55 (8, 123)	168 (68)	48.5 (12, 118)

There was no indication that mosquitoes responded to holes as an opportunity to pass through the netting. Many instances were recorded in both orientations in which mosquitoes flew across the hole, sometimes more than once, without passing through it or pausing. In other instances, mosquitoes were observed to rest on the netting immediately beside a hole, sometimes for many seconds, without seeming to respond to it. Irrespective of hole size, orientation or arena size, virtually all mosquitoes that passed through a hole did so while flying as opposed to by walking through. In many cases, hole passage was noted not to be clean; mosquitoes often bumped into the sides of the hole while passing through. Some mosquitoes bumped the holes’ edges several times appearing to ‘pinball’ through. Still others collided with the hole edges and failed to pass through.

**Fig. 4:**
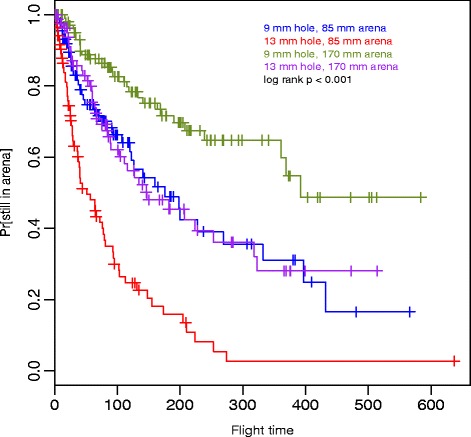
Kaplan-Meier survival functions comparing all combinations of hole and arena size irrespective of arena orientation

**Fig. 5:**
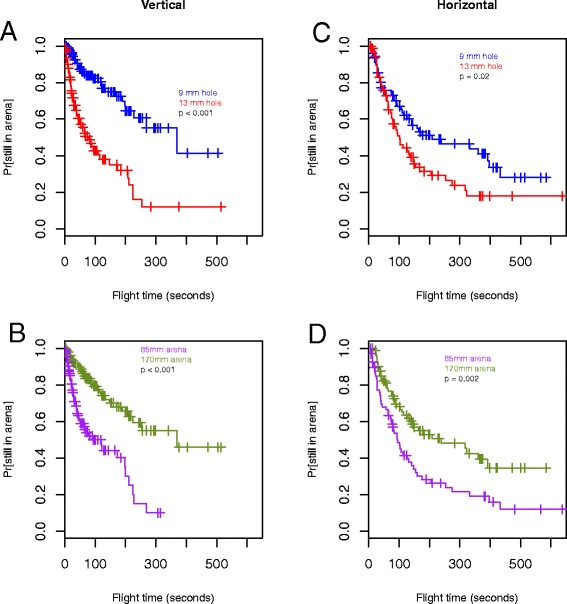
Kaplan-Meier survival functions comparing hole sizes (**a** and **c**) and arena sizes (**b** and **d**) stratified by arena orientation. (*p*-values from Cox PH models)

The rate of hole passage per second spent flying and walking was significantly higher for arenas with a 13 mm hole vs. arenas with a 9 mm hole (log rank *p*-value < 0.001) and for the small vs. large arenas (log rank *p*-value < 0.001). In order to compare all four arena configurations simultaneously, hole sizes and arena sizes were pooled to create one categorical variable with the following groups: 1) 9 mm hole, 85 mm arena, 2) 13 mm hole, 85 mm arena, 3) 9 mm hole, 170 mm arena, 4) 13 mm hole, 170 mm arena. Kaplan-Meier survival functions for each of the four configurations are shown in Figs. 4 and 5. Mosquitoes passed most frequently through 13 mm holes in the 85 mm arena and least frequently through 9 mm holes in the 170 mm arenas.

There was no significant interaction between arena size and hole size in either the horizontally oriented or the vertically oriented arenas. Both hole size and arena size were significant factors in determining the probability of passage for both orientations of the arenas (Fig. 5, Table 3). The rate of passage through a 13 mm hole was nearly four times the rate through a 9 mm hole at any given time during the observation period in vertically oriented arenas (Fig. 5a, Table 3, HR 3.96). Furthermore, mosquitoes in 85 mm arenas had more than three times higher passage rates than those in 170 mm arenas in the vertical orientation (Fig. 5b, Table 3, HR 3.43). Although these differences were not as pronounced in the horizontally oriented arenas, the larger hole size had a more than 60 % higher rate of passage than the smaller size hole (Fig. 5c, Table 3, HR 1.63), and the smaller arena diameter had nearly twice the rate of passage as the larger arena (Fig. 5c, Table 3, HR 1.94).

**Table 3:** Hazard ratio estimates from Cox PH models stratified by arena orientation

Experiment characteristics	N	Passed through hole (%)	HR (95 % CI)	*p-value*
Vertical orientation	219	79 (36)		
9 mm hole	123	28 (23)	Ref	
13 mm hole	96	51 (53)	3.96 (2.48, 6.33)	< 0.001
85 mm arena	110	50 (45)	3.43 (2.13, 5.51)	< 0.001
170 mm arena	109	29 (27)	Ref	
Horizontal orientation	151	89 (59)		
9 mm hole	74	49 (66)	Ref	
13 mm hole	77	40 (52)	1.63 (1.07, 2.49)	0.02
85 mm arena	82	39 (48)	1.94 (1.27, 2.95)	0.002
170 mm arena	69	50 (72)	Ref	

### Ambient conditions in arenas

**Fig. 6:**
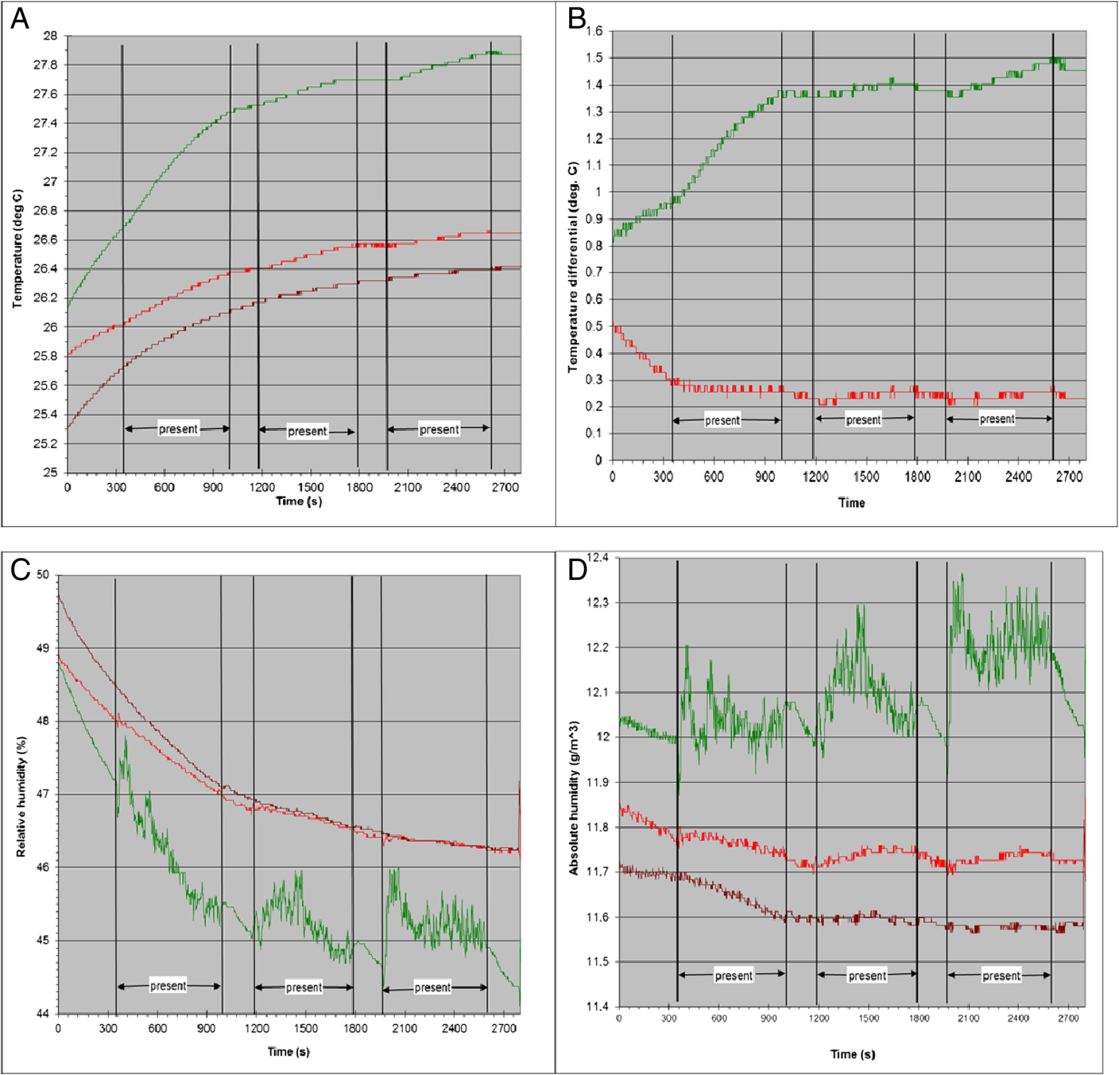
Temperature and humidity profiles at three positions in a simulated bed net. In all panels, the overhead position is green, the position to the side is red and the reference (room) is magenta. Experimenter occupied the simulated bed net in periods labelled ‘present’. **a**. Temperature, **b**. Temperature differential with the reference position, **c**. Relative humidity, **d**. Absolute humidity

Temperature at all measured locations generally increased from the start of the recordings (time zero) to the end (Fig. 6a) probably because the experimenter’s body heat was accumulating in the small well-insulated chamber. This increase was fastest in the overhead position of the simulated net even before the experimenter occupied it. Compared to the reference location (‘room’), temperatures were higher by an average of 0.2-0.3 °C at the side position of the simulated net and by 0.8°–1.5 °C at the overhead position (Fig. 6b). Overall, the temperature differential profile did not strongly reflect the presence of the experimenter in the simulated net at either the overhead or side positions. Relative humidity showed a decreasing trend from time zero at all locations because of the concurrently increasing temperature in the room. Relative humidity was strongly affected by the experimenter, being generally higher and more variable at the overhead position than at the side position when the experimenter was present (Fig. 6c). Absolute humidity when the experimenter was present in the simulated net was much higher and more variable at the overhead position than at the side position or at the room reference point (Fig. 6d).

## Discussion

### Activity in the simulated bed net

Levels of mosquito activity were significantly greater in arenas above the experimenter compared to arenas to the side. Measurements of temperature and relative humidity in both locations indicate that experimenter presence generated a convective plume that rose vertically, strongly affecting conditions in the overhead arenas but having very little effect on conditions in the arenas to the side of the experimenter. This would account for the greater activity levels exhibited by mosquitoes in the overhead arenas since moisture and heat plumes, and body and breath odours that would have been carried along with them, are known to be powerful mediators of close range host seeking in mosquitoes [15, 16]. Attractant or arrestant effects of some of these mediators likely explain the higher turning rates in flight paths seen in the overhead arenas. This pattern, in turn, may account for the significantly greater median pre-hole passage flight times in mosquitoes in overhead arenas (Table 2) since these mosquitoes would have covered less of the net per unit of flight time. Despite this, hole encounter rates in terms of time spent flying in the differently oriented arenas appear comparable since the ratios of median pre-hole passage flight time to median total flight time in vertical arenas (40.0 s:48.5 s = 0.83) and horizontal arenas (75.0 s:93.0 s = 0.81) were very close.

Temperature in overhead arenas, while showing a steady overall increase relative to room temperature and to temperature in arenas to the side, only weakly mirrored the experimenter’s presence in the simulated net and may not, in itself, account for the high levels of activity of the mosquitoes in the overhead arenas. On the other hand, although its overall level dropped over time, relative humidity in overhead arenas was highly responsive to experimenter presence-absence in the simulated net. The precise roles that the heat-moisture-odour stimulus play in mediating the mosquito host seeking response is not known but Takken *et al*. [17], working with female *An. gambiae* in a two-choice wind tunnel, concluded that rising relative humidity exerts a positive effect on host seeking while steady or dropping relative humidity does not. In their experiments, however, air temperature was held constant while relative humidity was altered by adjusting the amount of moisture in the air stream. This means that when relative humidity was decreased, the total amount of moisture in the air (absolute humidity) also decreased. Thus, in their work it is not possible to separate effects of changes in relative humidity from changes in absolute humidity.

Electrophysiological studies of hygroreceptive sensilla in the stick insect, *Carausius morosus*, show that these sensory organs have ‘wet’ and ‘dry’ cells whose firing frequencies change in opposite directions in response to changes in humidity thus amplifying the effect of small deviations [18]. When temperature and relative humidity values are combined to yield absolute humidity values in the arenas (Fig. 6d), it can be seen that, despite dropping relative humidity levels (due to increased temperature), the presence of the experimenter resulted in a net addition of moisture to the plume and in a sustained ‘noisy’ absolute humidity signal. If mosquito hygroreceptors work like those of the stick insect, the humidity signal likely contrasts strongly with background providing the mosquito with a strong indication of the presence of a potential host.

These are the first measurements of the heat and humidity conditions produced by the human body in a situation that can be compared to a bed net. While these measurements were taken in a simulated bed net, human occupant(s) probably create similar conditions around real bed nets. If so, this would confirm the speculations of several authors [19–22] that large numbers of host seeking mosquitoes are often observed on the tops of bed nets as the result of a convective plume from the net occupants. A thorough mapping of the conditions around bed nets and of the factors that affect these conditions (e.g. number and locations of sleepers, cross draughts, bed net size, proximity to walls, etc.) could provide important insights for ITN design and optimal deployment.

### Effects of hole size, arena size and orientation on hole passage

#### Hole passage behavior

In observations of mosquitoes in arenas, hole passage in both orientations happened in flight and not while walking. The expectation that hole passage probability in these experiments would be a positive function of hole area, and the resulting prediction that the rate of passage would be greater for the 13 mm holes than for the 9 mm holes was confirmed when the data were pooled across orientation (Table 3). This supports the conclusion that hole passage is, at least in part, a function of the chance of the mosquito encountering the hole while flying. This conclusion is further supported by the fact that the passage rate through holes of a given size (pooled across orientations) was higher in the smaller arenas than larger ones (Table 3), since smaller arenas presented less search area. These findings do not support the conclusion that hole passage was the result of the mosquito seeing, or otherwise detecting and orienting to, net holes.

### Passage through vertically-oriented holes-evidence for an edge effect

The ratio of the areas of the two hole sizes in these experiments was 2:1 (area of 13 mm diameter hole = 133 mm^2^, area of 9 mm hole = 64 mm^2^) leading to the prediction that hole passage rate in a given arena size would be about two times greater for the 13 mm diameter compared to the 9 mm diameter holes. This was the case for the horizontal orientation (Table 3, HR 1.63) but not in the vertical orientation (Table 3, HR 3.96); that is, in the vertical orientation passage rate was about 50 % lower through the 9 mm holes than expected on the basis of hole area alone. From this we conclude that there is a factor in addition to hole area affecting passage through holes in the vertical orientation and that this factor applies more to the smaller holes than to the larger holes.

The additional factor affecting hole passage could be interactions of the flying mosquito with the hole edge (i.e. a ‘hole edge’ effect). As previously noted, mosquito encounters with holes in the netting sometimes involved the mosquito colliding with the hole edges. While detailed information was not recorded, collisions ranged from direct hits to glancing contacts. It seems likely that the degree and number of these collisions would have an effect on hole passage.

**Fig. 7:**
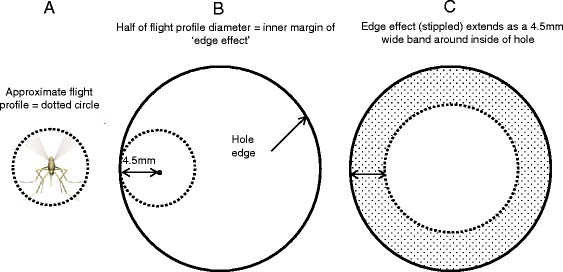
Hypothesized relationship of mosquito flight profile to edge effect. **A**. Approximate extent of in-flight profile diameter (9 mm) marled by dotted line. **B**. Centre of in-flight profile positioned one radius from the hole edge. **C**. Extent of 4.5 mm wide edge effect (stippled area) inside hole edge

In flight, the mosquito’s head, mouthparts, wings and legs extend outward partly occupying a volume referred to here as the ‘in-flight profile’. The diameter of the in-flight profile of *An. gambiae* can be estimated from high definition photographs of flying females of this species in Dickerson *et al.* [23]. In their Fig. 1, mosquitoes from the same colonies as those used in these experiments are shown in flight alongside 3 mm diameter water droplets. The in-flight profile diameter no doubt varies somewhat depending on the particular cross-section taken through it but it can be estimated from this as being about 9 mm (Fig. 7).

**Fig. 8:**
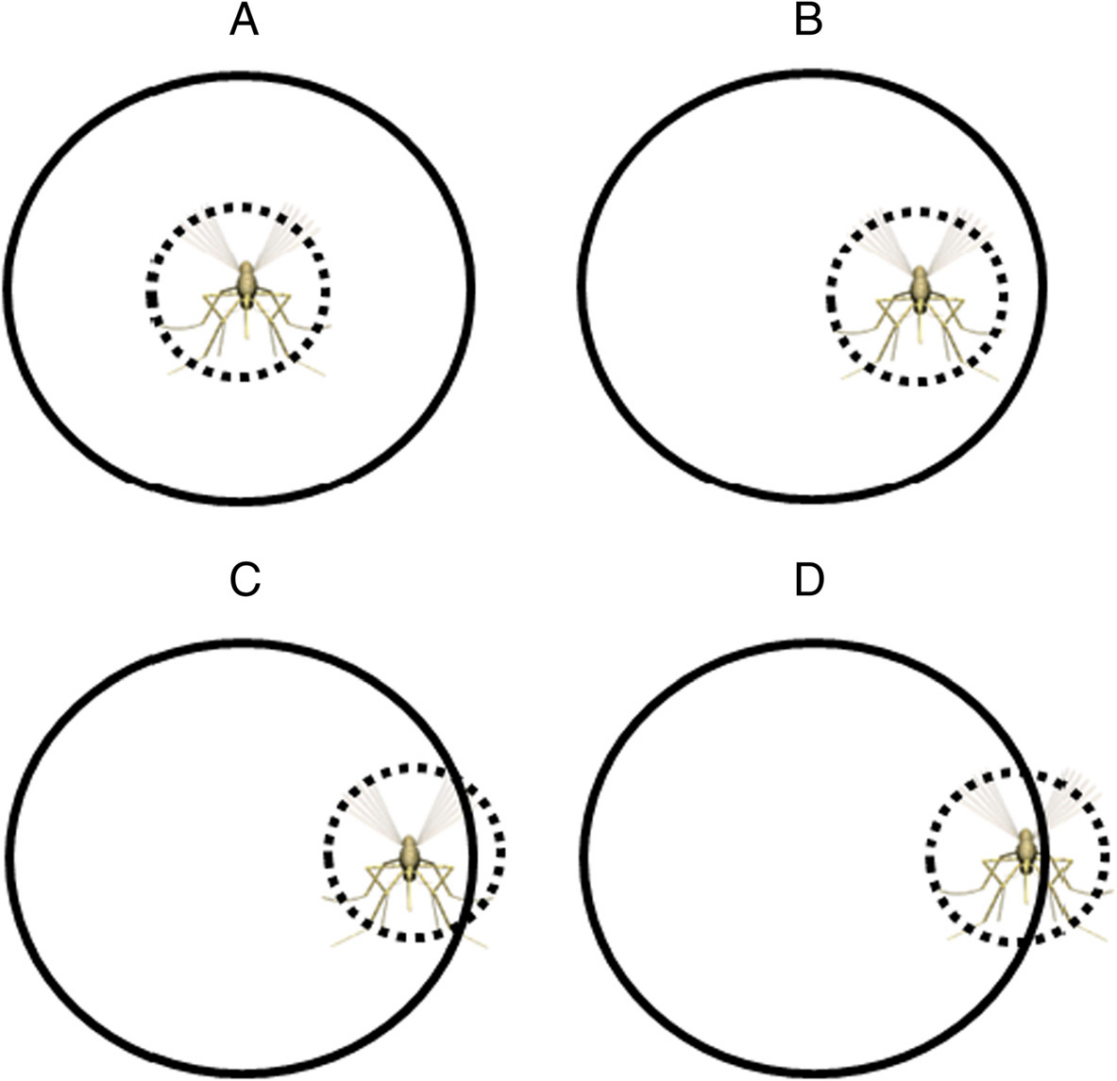
In-flight profile (dotted circle) at various possible positions relative to the hole edge (solid circle) and surrounding netting. **a**. and **b**. Hole passage unhindered because in-flight profile does not intersect hole edge or netting. **c**. Hole passage somewhat hindered because of a slight interaction with the hole edge and netting. **d**. Hole passage greatly hindered because of extensive interaction with hole edge and netting

If, when it is close to a hole in the netting, no part of the flying mosquito contacts the hole edge (i.e. if the center of the 9 mm in-flight profile is 4.5 mm or more from the hole edge (Fig. 8a,b)), there will be no collision and its passage through the hole would not be hindered by the edge effect. On the other hand, if the centre of flying mosquito’s in-flight profile is less than 4.5 mm from the hole edge, contact with the edge will occur and the probability of hole passage should be reduced in proportion to the amount of contact that occurred (Fig. 8c,d). Thus, a proportion of any hole’s area should be made less passable for the mosquito by the edge effect. This effect should account for increasingly large proportions of the hole area as hole diameter decreases. Conversely, the proportion of the hole’s area made less passable by the edge effect should decrease with increasing hole diameter.

This proposed edge effect could account for the approximately 50 % less-than-predicted 9 mm hole passability in vertically-oriented arenas since virtually every potential passage through a hole this size (by mosquitoes this size) will bring some part of the mosquito into contact with the hole edge. This effect would rapidly diminish as hole diameter increases but may be passage-limiting for holes much smaller than the 9 mm diameter holes tested here.

### Passage through horizontally-oriented holes

The fact that that comparative passage rates through 9 mm and 13 mm holes in horizontally oriented arenas (above the experimenter) were not significantly different from what they should have been based on area alone suggests there are orientation-specific factors that counteract the edge effect seen in vertically-oriented arenas. The convective plume passing from the experimenter through the overhead arenas, but not arenas to the side (Fig. 6), may be this counteracting factor. If the plume passed more easily through the holes in the overhead arena netting than through the intact net, this may have influenced mosquitoes to stay closer to holes thus increasing the probability of encountering the hole. The plume passing more readily through the hole than the intact netting may also, or alternatively, have provided orienting information that allowed more precise flight control (and, therefore, fewer collisions with the hole edges) during passage through the overhead holes. It may also be easier for reasons of in-flight profile geometry for a mosquito to pass through the overhead holes by dropping through vertically and downward passage is also likely to have been gravity-assisted.

### Implications for effects of hole shape

These experiments were done using only round holes but their results have implications for the passability of non-round holes which, it is well-documented, make up the majority of holes in bed nets. Smith *et al.* [10] report that small holes in ITNs start as small snags that, due the weave pattern of the netting material, gradually ravel creating oval or spindle-shaped holes. Other holes, such as those caused by chewing animals or by burns from candles and lanterns inside or near the net, may have highly irregular shapes. Long slender shapes and irregular shapes of a given area have larger edge to area ratios than round shapes of the same area (circles have the minimum circumference:area ratio of all geometric shapes). Thus, the edge effect may reduce passability of irregularly shaped holes even though they have a large area. In some cases, parts of irregular holes may be made effectively impassable by the edge effect.

### Implications for the assessment of physical bed net integrity

The WHOPES-recommended proportionate hole index (pHI) method for assessing bed net damage [24] groups holes on nets into four categories by size (estimated diameter). These categories are: 1) less than 0.5 cm, 2) 0.5 cm to 2 cm (‘finger’ size), 3) greater than 2 cm to 10 cm (‘fist’ size) and 4) greater than 10 cm (‘head’ size). In the pHI calculation, holes in category 1 are ignored because they are considered impassable while holes that fall into each of the other three categories are counted and each is assigned the diameter that corresponds to the mid-point of each category size range (e.g. all category 2 holes are assigned a diameter of 1.25 cm irrespective of actual size). Assigned hole areas for each size class are then multiplied by the number of holes in the size class and the products are added to yield the pHI. If results in this study translate to real bed nets they suggest that the pHI method has several shortcomings that could lead to significant error and inconsistency in bed net assessment. For instance, these results suggest that the passability for holes at the extremes of the ‘finger’ range on the net roof may differ by a factor of about 16 (based on relative areas of 0.5 cm and 2.0 cm diameter holes). In other words, for every mosquito entering the net through a 0.5 cm diameter hole in the roof, on average, 16 will enter through a 2 cm diameter hole. Despite this, the pHI would not differentiate between these in terms of entry risk. The range for ‘finger size’ holes should be even greater on the net sides where both area and the edge effect have an influence. Data from this study suggest that passability range for such holes may be two or three times greater than for roof holes (i.e. 30–40 times). Again, the pHI would assess the risk represented by these situations as the same. By the same reasoning, the ‘fist’ and ‘head’ hole size ranges also likely represent widely different entry risks that the pHI does not recognize.

### Importance of very small holes

The increasing impact of the edge effect on vertically oriented holes approaching 0.5 cm in diameter suggests that mosquitoes will not be able fly through holes around this size if they are on the sides of the bed net. The WHOPES-recommended practice of not counting holes smaller than 5 mm in ITN assessments is supported by this though only for holes in the net sides and ends. These results suggest that the passability of smaller holes on the bed net roof is not affected in the same way and they may be more passable than their counterparts on the net sides.

Work by Itoh *et al.* [25] also addresses bed net entry through very small holes. They observed that female *Culex pipiens pallens* readily flew through 1.6 cm X 1.6 cm holes in netting placed across a wind tunnel downwind of a mouse bait. However, if the holes were 0.8 mm X 0.8 mm or less, mosquitoes would land on the netting and would walk/squeeze through them. In their experiments, the netting was oriented vertically and the air stream passed through it horizontally after passing over the mouse. This is equivalent to the situation on the bed net where the air stream (convective plume) rises from the net occupant and passes through the horizontally oriented net roof. Itoh *et al.* concluded that landing occurred if the mosquitoes’ wings touched the hole sides. Video observations show that *An. gambiae* in the present study tolerated considerable contact with the hole edges and continued to fly but when the edge effect effectively closes small holes to passage through flight, this species too might resort to landing and walking/squeezing through small holes on the net roof.

## Conclusion

Mosquitoes respond to occupied bed nets in complex ways that are the result of their innate host seeking behaviours and the environment around the net. An improved understanding of this complex interaction is needed to inform changes in bed net design and deployment and more accurate ways of assessing the risk posed by bed nets in various states of deterioration.

Work by Lynd and McCall [20] and Sutcliffe and Yin [22] shows how mosquito pressure is distributed across the occupied bed net. The work reported here complements this by showing how mosquitoes are likely to respond to net damage when they encounter it on different areas of the net. Specifically, this study shows that the risk of mosquitoes getting into a bed net once they encounter a damaged area is probably determined by a combination of the extent of the damage (hole size), the shape of the damage (edge effects), and the orientation of the damage (whether it is on the roof or sides). In turn, this comes under the influence of the convective plume produced by the net occupant(s). More work is planned in these areas but, in particular, in describing extent of the edge effect and its influence on the passability of irregularly shaped holes and in understanding factors that influence the strength and configuration of the convective plume (e.g. number of net occupants, cross draughts in the room, etc.)

## Abbreviations

ITN: Insecticide-treated bed net
CO_2_: Carbon dioxide
CDC: United States Centers for Disease Control and Prevention
RH: Relative humidity
pHI: Proportionate hole index
